# Review of ‘BipolART’ by D. N. Wheatley (Springer, 2012)

**DOI:** 10.1186/1742-4682-10-2

**Published:** 2013-01-03

**Authors:** Paul S Agutter

**Affiliations:** 1 Theoretical Medicine and Biology Group, 26 Castle Hill, Glossop, Derbyshire, SK13 7RR, UK

## 

This delightful book comprises just 121 pages of main text, but in that short compass Denys Wheatley manages not only to convey much about bipolar disorder from the sufferer’s point of view but also to provoke the reader into pondering the nature of creativity in art and science. At the same time he contrives to entertain and to please the eye. Wheatley draws in part on his long personal experience of extreme mood swings, making stimulating observations about the value of art (particularly doodling) as therapy, and in part on his profound and wide-ranging scientific knowledge and his love of great paintings and music. The quality of his art work is well above ‘amateur’ standard, and the often abstract forms show the influence of his decades of research in zoology and cell biology, a fascination with geometry, the visual environment, and Europe’s cultural heritage. In addition to the reflective chapters and the analytical comments on individual drawings and paintings, the author gives us a fascinating stage-by-stage guide to the process by which a doodle can grow from insignificant beginnings into an eye-catching piece of art. He almost tempted me to take up doodling, but I doubt whether I could create any end-product to match his.

It would have been all too easy for a book of this kind, the personal reflections of a ‘manic-depressive’, to degenerate into navel-gazing and self-pity. But not for a single sentence does ‘BipolART’ commit that literary sin. Once I started to read the book I couldn’t put it down; only the irresistible urge to linger on individual pictures, relishing their beauty and intricacy, retarded my progression from first page to last. There are a few minor editing lapses – typographical errors and grammatical infelicities – but they impaired neither my understanding nor my enjoyment. This is a book that could be read for both pleasure and profit by psychologists, psychiatrists, art critics and philosophers interested in creativity, but above all by the interested non-specialist – the ‘man on the Clapham omnibus’, as Wheatley himself puts it. I recommend it warmly.

## Competing interests

The author declares no conflict of interest.

